# Reduced-Port Laparoscopic Distal Gastrectomy in Patients Aged ≥ 75 Years Versus <75 Years: Comparable Surgical Outcomes and Higher Medical Complication Events

**DOI:** 10.3390/medicina62040651

**Published:** 2026-03-29

**Authors:** Sung Kyu Kim, Ho Goon Kim

**Affiliations:** 1 Department of Surgery, Chonnam National University Hospital, Gwangju 61469, Republic of Korea; ribery12@naver.com; 2 Department of Surgery, Chonnam National University Medical School, Gwangju 61469, Republic of Korea

**Keywords:** stomach neoplasm, laparoscopy, gastrectomy, aged, safety

## Abstract

*Background and Objectives:* This study aimed to evaluate the safety and feasibility of reduced-port laparoscopic distal gastrectomy (RPLDG) in elderly patients. *Materials and Methods*: Electronic medical records of 226 patients who underwent RPLDG performed by a single high-volume surgeon at a single institution (Chonnam National University Hospital) between January 2015 and April 2020 were retrospectively analyzed. Among these patients, 60 were aged ≥ 75 years (elderly group), and 166 were younger than 75 years (non-elderly group). Patient characteristics, surgical outcomes, and short-term postoperative outcomes were compared between the two groups. *Results:* Surgical outcomes were comparable between the age groups. However, medical complication events, assessed using an event-based approach allowing multiple events per patient, were more frequent in patients aged ≥ 75 years. Compared with the non-elderly group, the elderly group demonstrated a higher frequency of overall postoperative complication events (18 [30%] vs. 29 [18%], *p* = 0.040), primarily attributable to medical complications (9 [15%] vs. 6 [4%], *p* < 0.01). The elderly group also showed a delayed time to first gas passage (3.5 [3.0–4.0] vs. 3.0 [3.0–3.0] days, *p* < 0.001). However, no statistically significant differences were observed in length of hospital stay (7.0 [6.0–10.0] vs. 6.0 [6.0–8.0] days, *p* = 0.262) or intraoperative blood loss (*p* = 0.831). No significant differences were found in surgical complication events (*p* = 0.05) or Clavien–Dindo grade ≥ 3 complication events (*p* = 0.13). In the risk factor analysis for complications, univariate analysis identified age ≥ 75 years as a significant factor. However, in the multivariate analysis, only respiratory comorbidities, liver disease, and poor ECOG performance status remained independent risk factors, whereas age ≥ 75 years was no longer statistically significant (*p* = 0.193). The finding regarding liver disease should be interpreted with caution because of the extremely small sample size. *Conclusions:* RPLDG appears to be a viable surgical option for patients aged ≥ 75 years, demonstrating acceptable surgical outcomes and severe complication rates comparable to those observed in non-elderly patients.

## 1. Introduction

Gastric adenocarcinoma is the world’s fifth most prevalent cancer and the third leading cause of cancer-related mortality, accounting for nearly 800,000 deaths globally [1]. In recent years, the average age at diagnosis in South Korea has declined, largely due to increased emphasis on population-based screening programs. Despite this trend, the highest incidence of gastric cancer still occurs among individuals in their seventh decade of life [2]. At the same time, South Korea is experiencing rapid population aging, and the associated increase in life expectancy is expected to further increase the incidence of gastric cancer among older adults. Nevertheless, specific treatment guidelines for gastric cancer in this elderly population have not yet been established.

Since its introduction in 1994 [3], laparoscopic-assisted distal gastrectomy has become a widely accepted surgical approach for early gastric cancer. Efforts to further reduce surgical invasiveness have focused on eliminating mini-laparotomy windows and decreasing the number of laparoscopic ports. As a result, several studies have demonstrated the feasibility and safety of reduced-port laparoscopic distal gastrectomy (RPLDG) in various patient populations, including individuals with obesity [4,5,6,7,8,9,10]. Although conventional laparoscopic gastrectomy has been established as a safe procedure for elderly patients, the feasibility of RPLDG in this population remains insufficiently investigated. While clinical evidence is gradually accumulating, studies focusing specifically on the oldest-old (e.g., patients aged 75 years and older) remain limited. At our institution, conventional five-port laparoscopic gastrectomy was initially adopted for the surgical management of gastric cancer. However, over the past decade, reduced three-port laparoscopic gastrectomy has been routinely performed in all patients with gastric cancer, regardless of sex, age, or clinical status. Therefore, the present study aimed to evaluate surgical outcomes and assess the feasibility of RPLDG in elderly patients with gastric cancer.

## 2. Materials and Methods

### 2.1. Patients

**Figure 1:**
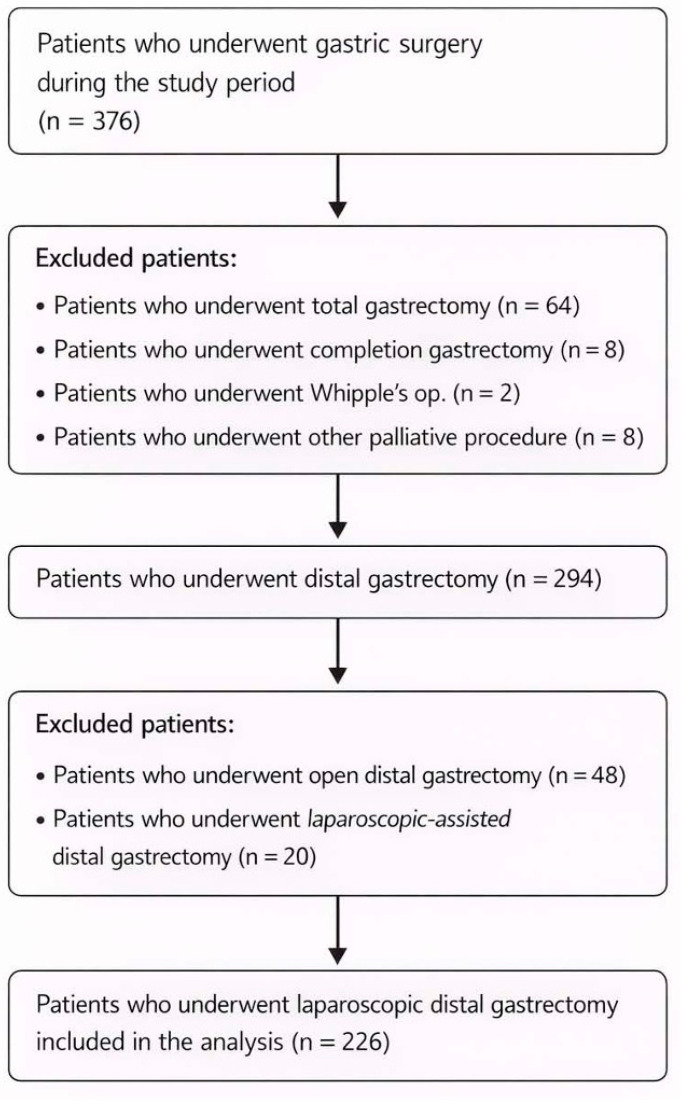
Flow diagram of patient selection for reduced-port laparoscopic distal gastrectomy.

A total of 226 patients with gastric adenocarcinoma who underwent Reduced-Port Laparoscopic Distal Gastrectomy at Chonnam National University Hospital between January 2015 and April 2020 were retrospectively analyzed. During the study period, consecutive patients surgically treated for gastric cancer were screened. Patients who did not receive a distal gastrectomy were excluded. Among the remaining cohort, those who underwent open or laparoscopy-assisted distal gastrectomy were further excluded. The detailed patient selection process is illustrated in Figure 1.

At our institution, RPLDG was primarily indicated for patients with clinical stage T1–2N0 gastric cancer. The cohort also included patients who required subsequent radical gastrectomy because of non-curative findings after endoscopic submucosal dissection (ESD), such as submucosal invasion, lymphovascular invasion, or positive resection margins [11]. The clinical T stage before ESD was determined based on gross endoscopic findings by experienced gastroenterologists. Endoscopic ultrasonography was routinely performed to evaluate tumor invasion depth and assess eligibility for ESD. When nodal metastasis was suspected, patients were informed of the risks and benefits of open versus laparoscopic surgery. For patients who elected laparoscopic surgery, RPLDG was also performed in selected cases with clinical stage T1–3N1 disease. During the study period, all patients who underwent RPLDG within these institutional indications were consecutively included regardless of age. All procedures were performed by a single surgeon who had completed more than 400 cases of conventional five-port laparoscopic gastrectomy at the time of study initiation.

According to the classification proposed by Ouchi et al. [12], patients aged ≥ 75 years were categorized as the elderly group, whereas those younger than 75 years were categorized as the non-elderly group. The two groups were compared with respect to short-term surgical outcomes, including operative results, hospital course, morbidity, and mortality.

All cases of gastric cancer were diagnosed by esophagogastroduodenoscopy with biopsy. Patients with confirmed gastric adenocarcinoma underwent preoperative imaging for clinical staging. Clinical stage was primarily evaluated using abdominal computed tomography (CT). When lymph node metastasis was suspected on CT, positron emission tomography–CT was additionally performed to assess distant metastasis. Comprehensive medical history taking and routine preoperative examinations, including chest radiography, electrocardiography, and laboratory tests, were performed in all patients to evaluate comorbidities and nutritional status. The extent of lymph node dissection and pathological staging were determined according to the Japanese Gastric Cancer Treatment Guidelines and the 8th edition of the Union for International Cancer Control/American Joint Committee on Cancer (UICC/AJCC) classification system [11,13]. Postoperative complications were assessed using the Clavien–Dindo classification and categorized as medical or surgical events according to the K-QIPS program criteria. Surgical complications included abdominal bleeding, ascites, gastric stasis, ileus, intra-abdominal infection, wound complications, and pancreatitis. Medical complications included cardiovascular, pulmonary, and renal events [14,15].

### 2.2. Surgical Procedure

The procedure was performed with the patient in the reverse Trendelenburg position. The camera assistant stood between the patient’s legs, and the surgeon stood on the patient’s right side. Laparoscopic port placement included a 12 mm camera port in the supraumbilical region, a 5 mm trocar in the right subcostal region, and a 12 mm trocar in the right lower quadrant, forming a mirrored inverted J-shaped configuration. To facilitate exposure and dissection of the lesser omentum, nylon sutures and Hem-o-lok clips were applied to the gastrohepatic ligament to provide liver traction.

Gastrectomy and regional lymph node dissection were performed in accordance with the Japanese Gastric Cancer Treatment Guidelines [11]. In most cases, D1+ lymph node dissection was performed; however, D2 dissection was undertaken in patients with suspected clinical stage T2 disease or lymph node metastasis. All procedures were performed using the same instruments used in conventional five-port gastrectomy, without additional devices for retraction or countertraction.

Lymph node dissection began with division of the greater omentum. This was followed by dissection of lymph node stations 5 and 6 after resection of station 4sb along the short gastric vessels up to the splenic hilum. The upper border of the pancreas was then carefully dissected while avoiding pancreatic compression. Dissection subsequently proceeded sequentially through stations 8, 12a, 11p, 9a, and 7.

The stomach was transected using an Endo GIA stapler (Signia™, Medtronic plc, Dublin, Ireland), and the specimen was retrieved through the extended umbilical port site using a laparoscopic retrieval bag. Reconstruction was performed intracorporeally in most patients using either Billroth-II or Roux-en-Y techniques. Billroth-I reconstruction was not performed because the presence of only two working ports on the patient’s right abdominal wall created an unfavorable stapling angle.

Among the available options, Billroth-II reconstruction was preferred in most cases because it is technically simpler and requires a shorter operative time, which helped minimize operative stress in this predominantly elderly patient population [16]. Although Roux-en-Y reconstruction is known to reduce reflux, a Braun anastomosis was selectively performed in a subset of relatively younger patients undergoing Billroth-II reconstruction to reduce potential biliary reflux while maintaining overall surgical efficiency.

### 2.3. Postoperative Care

A standardized clinical pathway based on the Enhanced Recovery After Surgery protocol was applied to all gastrectomy patients. Early ambulation and water intake were initiated on postoperative day (POD) 1. Oral feeding began with a liquid diet on POD 2 and was advanced to a soft diet on POD 3. Discharge was routinely recommended on POD 6 for patients who met the following criteria: absence of abnormal physical findings, normal laboratory results, adequate pain control without the need for intravenous or oral analgesics, no significant gastrointestinal symptoms, and tolerance of oral intake. In some cases, discharge was delayed because of individual patient circumstances or personal requests.

### 2.4. Data Collection

Patient data were obtained from electronic medical records. The study protocol was approved by the Institutional Review Board of Chonnam National University Hospital, Gwangju, South Korea, which waived the requirement for informed consent because of the retrospective study design (No. CNUH-2023-282). Clinicopathological variables, including age, sex, body mass index (BMI), American Society of Anesthesiologists (ASA) physical status score, surgical history, laboratory findings, and tumor–node–metastasis (TNM) stage, were prospectively collected. Surgical variables, including operative procedure, operative time, intraoperative blood loss, and the total numbers of retrieved and metastatic lymph nodes, were also recorded. Postoperative outcomes assessed included time to first flatus, time to resumption of oral intake, length of hospital stay, and postoperative complications, which were categorized as medical or surgical. Postoperative morbidity and mortality were defined as any complication or death occurring within 30 days after surgery or during the index hospitalization. Complications were recorded and analyzed as events rather than as patient-level rates, with multiple complications occurring in a single patient counted as separate events. The severity of postoperative complications was graded according to the Clavien–Dindo classification [14], and pathological staging was determined based on the 7th edition of the Union for International Cancer Control/American Joint Committee on Cancer (UICC/AJCC) TNM classification system [13].

### 2.5. Statistical Analysis

All statistical analyses were performed using IBM SPSS Statistics software, version 27.0 (IBM Corp., Armonk, NY, USA). The normality of continuous variables was assessed using the Shapiro–Wilk test. Because the continuous variables did not follow a normal distribution, they were presented as medians with interquartile ranges (IQRs) and compared between groups using the Mann–Whitney U test. Categorical variables were expressed as frequencies and percentages and were compared using the chi-square test or Fisher’s exact test, as appropriate. To identify risk factors for postoperative complications, univariate analysis was initially performed. Variables with a *p*-value < 0.1 in the univariate analysis were subsequently entered into a multivariate logistic regression model. The results were reported as odds ratios (ORs) with 95% confidence intervals (CIs). A *p*-value < 0.05 was considered statistically significant.

## 3. Results

### 3.1. Patients’ Characteristics

Preoperative clinical characteristics of the elderly and non-elderly groups are summarized in Table 1. The median age was 78.0 years (IQR, 76.0–80.0) in the elderly group and 62.0 years (IQR, 54.0–69.0) in the non-elderly group (*p* < 0.001). In both groups, gastric cancer was more prevalent in men than in women (63.3% vs. 36.7% in the elderly group and 74.7% vs. 25.3% in the non-elderly group; *p* = 0.090). Despite the inclusion of one patient with an Eastern Cooperative Oncology Group (ECOG) performance status score of 4 in the non-elderly group, overall ECOG scores were significantly higher in the elderly group (*p* = 0.01). In addition, 90.0% of elderly patients had ASA physical status score ≥ 2, which was significantly higher than the 55.4% observed in the non-elderly group (*p* < 0.001).

Comorbid conditions were more prevalent in the elderly group than in the non-elderly group (88.3% vs. 63.3%, *p* < 0.001), with significant differences observed for hypertension (63.3% vs. 43.4%, *p* = 0.010) and respiratory diseases (18.3% vs. 6.0%, *p* = 0.010). The elderly group also demonstrated significantly lower levels of albumin (4.3 g/dL [IQR, 4.1–4.4] vs. 4.4 g/dL [IQR, 4.2–4.6]; *p* = 0.001) and hemoglobin (13.4 g/dL [IQR, 12.3–14.3] vs. 14.3 g/dL [IQR, 13.0–15.0]; *p* < 0.001) than the non-elderly group. However, no significant differences were observed between the groups in preoperative clinical stage (*p* = 0.830) or BMI (*p* = 0.314).

### 3.2. Surgical Outcomes

Surgical outcomes are summarized in Table 2. The median estimated blood loss was 45.0 mL (IQR, 20.0–60.0) in the elderly group and 40.0 mL (IQR, 30.0–60.0) in the non-elderly group, with no statistically significant difference between the groups (*p* = 0.831). The median operative time was also comparable between the groups: 210.0 min (IQR, 185.0–239.0) in the elderly group and 205.0 min (IQR, 180.0–230.0) in the non-elderly group (*p* = 0.922). No conversion to open surgery occurred in this study.

To maintain surgical safety in technically challenging cases, additional port placement (>3 ports) was required in 15.0% (9/60) of elderly patients and 18.7% (31/166) of non-elderly patients, with no significant difference between the groups (*p* = 0.520). R0 resection was achieved in all patients. Most patients underwent Billroth-II reconstruction (96.7% in the elderly group vs. 93.4% in the non-elderly group, *p* = 0.280). No statistically significant differences were observed between the groups in tumor location (*p* = 0.540), number of retrieved lymph nodes (*p* = 0.400), extent of lymph node dissection (*p* = 0.600), or pathological stage (*p* = 0.890).

### 3.3. Postoperative Short-Term Outcomes

Short-term postoperative outcomes are presented in Table 3. The elderly group experienced a higher frequency of overall postoperative complication events than the non-elderly group (18 [30.0%] vs. 29 [17.5%], *p* = 0.040), as well as a greater number of Clavien–Dindo grade ≥ 2 complication events (7 [11.7%] vs. 4 [2.4%], *p* < 0.010). When complications were categorized as medical or surgical, medical complication events occurred significantly more frequently in the elderly group (9 [15.0%] vs. 6 [3.6%], *p* < 0.010), whereas surgical complication events did not differ significantly between the groups (*p* = 0.050). Pulmonary complications accounted for more than half of the medical complications in both groups and were more frequent in the elderly group (6 [10.0%] vs. 3 [1.8%], *p* = 0.010). Renal complications were also more common in elderly patients (3 [5.0%] vs. 1 [0.6%], *p* = 0.030).

Regarding recovery indicators, the median time to first flatus was significantly longer in the elderly group than in the non-elderly group (3.5 days [IQR, 3.0–4.0] vs. 3.0 days [IQR, 3.0–3.0]; *p* < 0.001). The median length of hospital stay was slightly longer in the elderly group, although the difference was not statistically significant (7.0 days [IQR, 6.0–10.0] vs. 6.0 days [IQR, 6.0–8.0]; *p* = 0.262). No significant differences were observed between the groups in median time to diet commencement (2.0 days [IQR, 2.0–2.0] in both groups; *p* = 0.974), 30-day readmission, postoperative transfusion, or postoperative fever.

### 3.4. Risk Factors for Postoperative Complications

Univariate (Table 4) and multivariate (Table 5) logistic regression analyses were performed to identify independent risk factors for postoperative complications. In the univariate analysis, age ≥ 75 years (OR 2.025, 95% CI 1.023–4.005, *p* = 0.040), respiratory comorbidity (OR 4.128, 95% CI 1.633–10.435, *p* = 0.001), liver disease (OR 5.581, 95% CI 1.104–28.216, *p* = 0.037), and ECOG performance status (OR 2.923, 95% CI 1.488–5.742, *p* = 0.002) were significantly associated with an increased risk of overall complications.

In the multivariate model, which included clinically relevant variables and those with *p* < 0.1 in the univariate analysis, respiratory comorbidity (adjusted OR [aOR] 3.651, 95% CI 1.351–9.871, *p* = 0.011), liver disease (aOR 6.962, 95% CI 1.039–46.656, *p* = 0.046), and ECOG performance status (aOR 2.576, 95% CI 1.196–5.549, *p* = 0.016) remained significant independent risk factors. Although age ≥ 75 years was significant in the univariate analysis, it was not statistically significant in the multivariate model (aOR 1.640, 95% CI 0.778–3.455, *p* = 0.193).

## 4. Discussion

In 2015, individuals aged 60 years and older accounted for 12% of the global population. With an annual growth rate of 3.26%, the elderly population is expected to continue increasing in the short to medium term [17]. In light of these demographic trends, identifying surgical procedures that are both appropriate and safe for elderly patients has become increasingly important. Numerous studies have examined the safety of gastrectomy in elderly populations, demonstrating that both open and laparoscopic gastrectomy can be performed safely and feasibly in this group [18,19,20]. However, data regarding the safety of reduced-port surgery in geriatric patients remain limited. To our knowledge, this study is the first to evaluate the safety of reduced-port laparoscopic gastrectomy in elderly patients. Furthermore, unlike previous studies that used an age cutoff of 65 years, this study applied a threshold of 75 years, providing insight into surgical outcomes in a more advanced elderly population.

Most previous studies evaluating laparoscopic gastrectomy for gastric cancer have reported no meaningful differences in intraoperative blood loss between elderly and non-elderly patients, with some studies even describing lower blood loss in older cohorts [21,22,23,24,25,26,27,28]. The findings of the present study are consistent with this literature and suggest that reduced-port laparoscopic gastrectomy can be performed safely in elderly patients. Although older individuals are often considered to have a higher risk of bleeding because of tissue fragility or the use of antithrombotic therapy, careful surgical technique and adequate experience appear to mitigate these risks, allowing outcomes comparable to those observed in younger patients.

Ryu et al. [29] reported that vascular injury resulting in bleeding or organ ischemia represents one of the most serious complications during laparoscopic distal gastrectomy. They identified surgeon experience and the extent of lymph node dissection as key determinants of intraoperative bleeding. In the present study, surgical technique and procedural complexity were consistent between groups because all operations were performed by a single experienced surgeon using standardized operative methods. The comparable intraoperative outcomes observed between age groups therefore likely reflect procedural consistency rather than differences in patient age alone.

Previous studies have also shown that pneumoperitoneum during laparoscopic gastrectomy may worsen pre-existing respiratory conditions and contribute to postoperative pulmonary complications, including pneumonia, atelectasis, and pleural effusion. Cho et al. [30] reported that elderly patients with underlying respiratory disease experienced a higher incidence of postoperative respiratory complications after laparoscopic gastrectomy, and chronic obstructive pulmonary disease has similarly been identified as a risk factor for postoperative morbidity [31]. More recently, frailty-based risk stratification has been proposed as a useful framework for explaining these postoperative medical events. Sakurai et al. [32] demonstrated that a higher frailty index was independently associated with non-surgical-site complications following gastrectomy, particularly pneumonia. The present findings are consistent with these observations and suggest that compromised preoperative pulmonary physiology plays an important role in the development of postoperative pulmonary complications in elderly patients.

In addition, the optimal approach to preoperative risk assessment in elderly patients with gastric cancer remains an important clinical issue. Although ECOG performance status is widely used as a pragmatic measure of functional capacity, emerging evidence suggests that frailty-based assessments, such as comprehensive geriatric evaluation or frailty indices, may provide additional prognostic information in older patients undergoing gastrectomy [33]. These approaches may help shift clinical decision-making away from reliance on chronological age alone toward a more comprehensive assessment of physiological vulnerability and perioperative risk.

Postoperative complication events were more frequent in elderly patients; however, no meaningful differences were observed in surgical complications between the groups. The observed increase was primarily related to medical complications, particularly pulmonary and renal events. This pattern likely reflects the baseline physiological condition of elderly patients. In this cohort, elderly patients demonstrated poorer nutritional indicators and a higher prevalence of respiratory comorbidities, factors that may reduce physiologic reserve and increase susceptibility to postoperative medical events. Differences between the groups were limited to lower-grade complications according to the Clavien–Dindo classification, whereas severe complications requiring invasive intervention were comparable. These findings suggest that although elderly patients may experience more minor postoperative medical events, reduced-port gastrectomy maintains an acceptable safety profile with respect to serious surgical complications.

Delayed passage of first flatus was more frequently observed in elderly patients, a finding consistent with previous studies evaluating laparoscopic gastrectomy in older populations [34,35]. This observation likely reflects slower recovery of gastrointestinal motility in elderly individuals. Despite this delay, both groups followed the same postoperative clinical pathway, and the timing of dietary advancement remained comparable. Similarly, no meaningful differences were observed in the length of hospital stay. These findings indicate that with standardized perioperative management and careful postoperative monitoring, elderly patients can achieve postoperative recovery patterns similar to those of younger individuals.

A major strength of this study is the consecutive enrollment of all patients meeting the surgical indications, which may reduce potential selection bias often associated with retrospective cohorts. Furthermore, technical consistency was ensured because all procedures were performed by a single high-volume surgeon with extensive prior experience, thereby minimizing variability in surgical technique. In this study, RPLDG appeared to be a safe and feasible option for patients aged ≥ 75 years, with severe complication rates (Clavien–Dindo grade ≥ 3) comparable to those in younger patients. Furthermore, there was no statistically significant difference in intraoperative blood loss between the elderly and non-elderly groups, further confirming the technical safety and feasibility of the procedure in older patients.

A notable strength of this study is the consecutive inclusion of all eligible patients meeting the surgical criteria, which helps reduce potential selection bias commonly associated with retrospective analyses. In addition, procedural consistency was maintained because all operations were performed by a single experienced surgeon using standardized techniques. Under these conditions, RPLDG appeared to be a safe and feasible surgical option for elderly patients, with severe complication outcomes comparable to those observed in younger individuals.

Risk factor analysis further highlights the importance of physiological condition rather than chronological age in determining postoperative outcomes. Although older age was associated with complications in unadjusted analyses, multivariable modeling identified respiratory comorbidities, liver disease, and poor performance status as the primary independent predictors. These findings highlight that surgical risk in elderly patients is more closely related to underlying comorbidity burden and functional status than to age itself. The association with liver disease should be interpreted cautiously because of the small number of affected patients in this cohort. Nevertheless, these results emphasize the importance of careful preoperative evaluation and optimization of functional status when selecting elderly patients for reduced-port gastrectomy.

Despite the clinical relevance of these findings, several limitations should be acknowledged. This study was a single-center retrospective analysis, and the results may partly reflect surgeon-specific expertise, which may limit generalizability to other institutions and operators. In addition, because most patients had early-stage disease, caution is required when extrapolating these findings to older patients with more advanced gastric cancer.

Future research should validate these findings in multicenter cohorts to assess reproducibility across different practice settings and surgeons with varying levels of experience. Prospective studies incorporating standardized perioperative protocols and patient-reported outcomes may further clarify the clinical value and patient-centered benefits of reduced-port gastrectomy in this population. In addition, inclusion of patients with a broader range of tumor stages and comorbidity profiles may help refine surgical indications and improve risk stratification for older adults.

## 5. Conclusions

RPLDG is a viable surgical option for carefully selected elderly patients when performed by an experienced surgeon. Postoperative recovery and severe complication rates were comparable between age groups; however, the higher incidence of medical complications in elderly patients highlights the importance of careful preoperative assessment and optimization. Because most patients in this cohort had early-stage disease, additional multicenter studies are needed to confirm the broader applicability and oncological safety of this approach in more diverse elderly populations.

## Figures and Tables

**Table 1:** Baseline characteristics of patients undergoing reduced-port laparoscopic distal gastrectomy for gastric cancer.

Variables	Elderly (n = 60)	Non-Elderly (n = 166)	*p*-Values
Age (years)	78.0 (76.0–80.0)	62.0 (54.0–69.0)	<0.001
Gender			0.09
Male	38 (63%)	124 (75%)	
Female	22 (37%)	42 (25%)	
BMI (kg/m^2^)	23.7 (21.5–25.5)	23.9 (21.7–26.3)	0.314
ASA score			<0.001
1	8 (13%)	74 (45%)	
2	46 (77%)	83 (50%)	
3	6 (10%)	9 (5%)	
ECOG score			0.01
0	44 (73%)	137 (83%)	
1	13 (22%)	27 (16%)	
2	3 (5%)	1 (1%)	
3	0 (0%)	0 (0%)	
4	0 (0%)	1 (1%)	
Comorbid disease	53 (88%)	105 (63%)	<0.001
Cardiovascular	9 (15%)	20 (12%)	0.56
Hypertension	38 (63%)	72 (43%)	0.01
Diabetes mellitus	13 (22%)	39 (24%)	0.77
Respiratory	11 (18%)	10 (6%)	0.01
Cerebrovascular	5 (8%)	7 (4%)	0.22
Renal disease	1 (2%)	2 (1%)	0.79
Liver disease	1 (2%)	4 (2%)	0.74
Other	9 (15%)	17 (10%)	0.32
History of abdominal surgery	5 (8%)	14 (8%)	0.98
Endoscopic submucosal dissection	6 (10%)	30 (18%)	0.14
Clinical stage			0.83
Stage I	58 (97%)	160 (96%)	
Stage II	2 (3%)	5 (3%)	
Stage III	0 (0%)	1 (1%)	
Nutrition status			
Albumin (g/dL)	4.3 (4.1–4.4)	4.4 (4.2–4.6)	0.001
Hemoglobin(g/dL)	13.4 (12.3–14.3)	14.3 (13.0–15.0)	<0.001

Values are expressed as means ± standard deviation (SD) or n (%). Abbreviations: BMI, Body Mass Index; ASA, American Society for Anesthesiologists; ECOG, Eastern Cooperative Oncology Group.

**Table 2:** Intraoperative surgical outcomes of reduced-port laparoscopic distal gastrectomy in elderly patients.

Variables	Elderly (n = 60)	Non-Elderly (n = 166)	*p*-Value
Operation time (min)	210 (185.0–239.0)	205 (180.8–230.0)	0.922
Blood loss (mL)	45.0 (20.0–60.0)	40.0 (20.0–50.0)	0.831
Additional port placement (>3 ports)	9 (15%)	31 (19%)	0.52
Omentectomy			0.43
Bursectomy	2 (3%)	10 (6%)	
Complete	58 (97%)	156 (94%)	
Reconstruction			0.28
Billroth-II	58 (97%)	154 (93%)	
Roux-en-Y	2 (3%)	12 (7%)	
Lymph node dissection			0.60
D1+	32 (53%)	82 (49%)	
D2	28 (47%)	84 (51%)	
Number of retrieved lymph nodes	36 ± 17.5	37 ± 15.6	0.40
Combined resection	3 (5%)	12 (7%)	0.55
Curative			1
R0	60 (100%)	166 (100%)	
R1/R2	0 (0%)	0 (0%)	
Tumor size (mm)	23.1 ± 11.1	21.1 ± 11.2	0.74
Tumor location			0.54
U	2 (3%)	6 (4%)	
M	12 (20%)	45 (27%)	
L	46 (77%)	115 (69%)	
Depth of tumor invasion			0.72
T1	53 (88%)	151 (91%)	
T2	5 (8%)	8 (5%)	
T3	1 (2%)	5 (3%)	
T4	1 (2%)	2 (1%)	
Lymph node metastasis			0.55
N0	51 (85%)	150 (90%)	
N1	6 (10%)	9 (5%)	
N2	2 (3%)	3 (2%)	
N3	1 (2%)	4 (2%)	
Pathological stage			0.89
I	55 (92%)	153 (92%)	
II	4 (7%)	9 (5%)	
III	1 (2%)	4 (2%)	
Histological differentiation			0.76
Well-differentiated	23 (38%)	62 (37%)	
Moderately differentiated	19 (32%)	49 (30%)	
Poorly differentiated	18 (30%)	52 (31%)	
Others	0 (0%)	3 (2%)	

Values are expressed as means ± standard deviation (SD) or n (%). Abbreviations: U, Upper 1/3rd; M, Middle 1/3rd; L, Lower 1/3rd.

**Table 3:** Postoperative short-term outcomes and complications in the study cohort.

Variables	Elderly (n = 60)	Non-Elderly (n = 166)	*p*-Value
Overall complications	18 (30%)	29 (18%)	0.04
CD grade 1	5 (8%)	16 (10%)	0.77
Medical complication	1 (2%)	1 (1%)	0.45
Surgical complication	5 (8%)	15 (9%)	0.87
CD grade 2	7 (12%)	4 (2%)	<0.01
Medical complication	5 (8%)	2 (1%)	0.01
Surgical complication	6 (10%)	4 (2%)	0.01
CD grade ≥ 3	4 (7%)	4 (2%)	0.13
Medical complication	2 (3%)	2 (1%)	0.28
Surgical complication	3 (5%)	2 (1%)	0.09
Mortality	0 (0%)	0 (0%)	1
Postoperative complication			
Medical complication	9 (15%)	6 (4%)	<0.01
Cardiovascular	1 (2%)	1 (1%)	0.45
Pulmonary	6 (10%)	3 (2%)	0.01
Renal	3 (5%)	1 (1%)	0.03
Others	0 (0%)	2 (2%)	0.39
Surgical complication	16 (27%)	25 (15%)	0.05
Abdominal bleeding	0 (0%)	1 (1%)	0.55
Ascites	3 (5%)	4 (2%)	0.32
Gastric stasis	1 (2%)	9 (5%)	0.23
Ileus	6 (10%)	6 (4%)	0.06
Intraabdominal infection	4 (7%)	3 (2%)	0.06
Wound	1 (2%)	2 (1%)	0.79
Pancreatitis	2 (3%)	3 (2%)	0.49
Length of hospital stay (days)	7 (6–10)	6 (6–8)	0.262
Time to first flatus (days)	3.5 (3–4)	3 (3–3)	<0.001
Days to solid diet (days)	2 (2–2)	2 (2–2)	0.974
Transfusion	3 (5%)	4 (2%)	0.32
Fever	8 (13%)	15 (9%)	0.35
Re-admission within 30 days after surgery	1 (2%)	4 (2%)	0.74

Values are presented as mean ± standard deviation (SD) or as events, n (%). Abbreviation: CD, Clavien–Dindo classification. Complications were analyzed as events; multiple events per patient were permitted.

**Table 4:** Univariate analysis of factors associated with postoperative complications.

Factors	Comparison	All Cases (n = 226)	Complications (n = 47)	OR (95% CI)	*p*-Value
Age (years)	<75/≥75	166/60	29/18	2.025 (1.023–4.005)	0.040
	<70/≥70	130/96	24/23	1.378 (0.723–2.628)	0.329
	<65/≥65	101/125	20/27	1.102 (0.576–2.109)	0.769
Gender	Male/Female	162/64	33/14	1.095 (0.541–2.216)	0.802
Comorbidity	Overall	158	33		
	Hypertension	110	20	0.733 (0.383–1.401)	0.346
	Diabetes Mellitus	52	12	1.191 (0.566–2.507)	0.644
	Cardiovascular	29	5	0.769 (0.277–2.137)	0.613
	Respiratory	21	10	4.128 (1.633–10.435)	0.001
	Cerebrovascular	12	3	1.288 (0.334–4.965)	0.712
	Liver disease	7	4	5.581 (1.104–28.216)	0.037
	Renal disease	5	2	2.800 (0.450–17.433)	0.270
ASA score	1/≥2	82/144	20/27	0.715 (0.372–1.377)	0.315
	≤2/≥3	211/15	42/5	2.012 (0.653–6.200)	0.216
ECOG state	0/≥1	181/45	30/17	3.056 (1.489–6.271)	0.002
	≤1/≥2	221/5	45/2	2.607 (0.423–16.075)	0.285
	≤2/≥3	225/1	47/0	0.994 (0.984–1.005)	0.608
Estimated blood loss	<40/≥40	118/108	25/22	0.952 (0.500–1.811)	0.88
LN dissection	≤D1+/≥D2	114/112	22/25	1.202 (0.631–2.287)	0.576
Tumor stage	EGC/AGC	219/7	46/1	0.627 (0.074–5.337)	0.666

OR, odds ratio; CI, confidence interval; ECOG, Eastern Cooperative Oncology Group.

**Table 5:** Multivariate analysis of factors associated with postoperative complications.

Factors	B	S.E.	Wald	OR Value	95% CI	*p*-Value
Age	0.495	0.38	1.691	1.64	0.778–3.455	0.193
respiratory	1.295	0.507	6.514	3.651	1.351–9.871	0.011
renal	1.664	1.374	1.467	5.282	0.357–78.050	0.226
liver disease	1.94	0.971	3.997	6.962	1.039–46.656	0.046
ECOG state	0.946	0.392	5.838	2.576	1.196–5.549	0.016

B, unstandardized regression coefficient; S.E., standard error; Wald, Wald chi-square statistic; OR, odds ratio (adjusted odds ratio in multivariate analysis); 95% CI, 95% confidence interval; *p*-value, significance probability.

## Data Availability

The data presented in this study are available from the corresponding author upon reasonable request.
